# Chronic heat-induced discoid lupus erythematosus

**DOI:** 10.1016/j.jdcr.2026.03.057

**Published:** 2026-04-08

**Authors:** Sara Araghi, Christine T. Pham, Sophia Ederaine, Kenneth Linden

**Affiliations:** Department of Dermatology, University of California, Irvine, Irvine, California

**Keywords:** discoid lupus erythematosus, heat, koebner phenomenon, lupus

## Introduction

Discoid lupus erythematosus (DLE) has been associated with several triggers, including ultraviolet radiation, medications, smoking, and viral infections.^1^ Lesions typically appear in photodistributed areas such as the face and head. The Koebner phenomenon, characterized by the formation of new lesions at sites of trauma, is commonly observed in conditions like psoriasis or lichen planus.^2^ Although rarely reported, the Koebner phenomenon has also been associated with some cases of cutaneous lupus, including DLE. Additionally, heat-induced Koebner phenomenon occurs when heat trauma triggers lesion formation through inflammatory processes specific to the underlying condition. Sources include radiators, heating pads, cooking equipment, and sun exposure.^3^ Although heat-induced Koebner phenomenon has been infrequently reported in cutaneous lupus erythematosus, we present a rare documented case of DLE induced by use of a heating blanket.

## Case report

**Fig 1:**
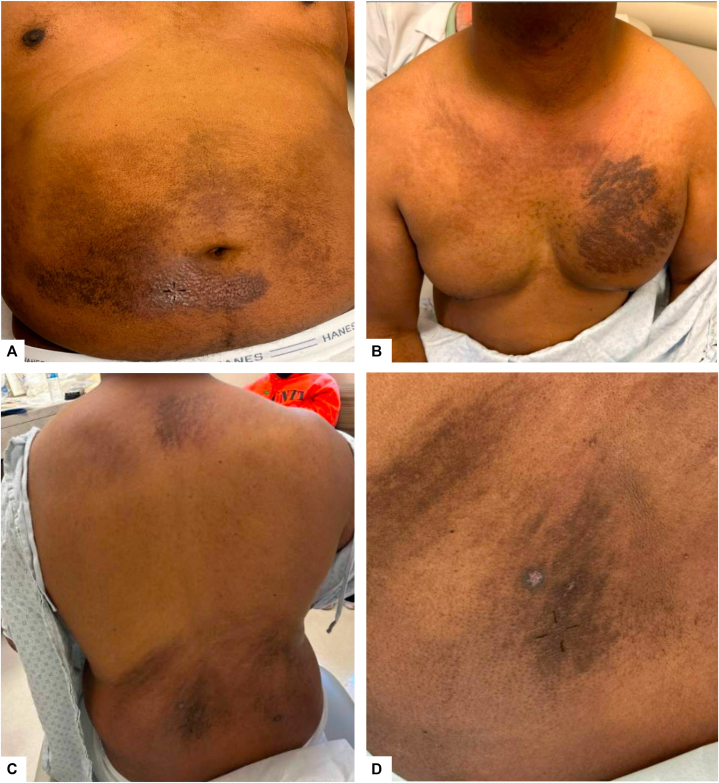
**A-D,** 54-year-old man with hyperpigmented, scaly linear, and papular plaques on the left chest, flanks, lower back, right lower abdomen, and knees. Biopsy sites were obtained on the **(A)** right periumbilical and **(D)** left lower back.

A 54-year-old male presented with a 6-week history of a pruritic rash on his chest, back, and knees. Medical history includes hypertension, hyperlipidemia, and a 5-year history of DLE lesions exclusively on face and scalp, treated with oral hydroxychloroquine, anifrolumab infusions, and topical ruxolitinib and clobetasol cream. He had never met criteria for systemic lupus erythematosus (SLE). Other pertinent history included nightly use of a heating blanket. Dermatologic examination revealed hyperpigmented, scaly linear and papular plaques on the left chest, flanks, lower back, right lower abdomen, and knees (Fig 1). Punch biopsies were performed.

**Fig 2:**
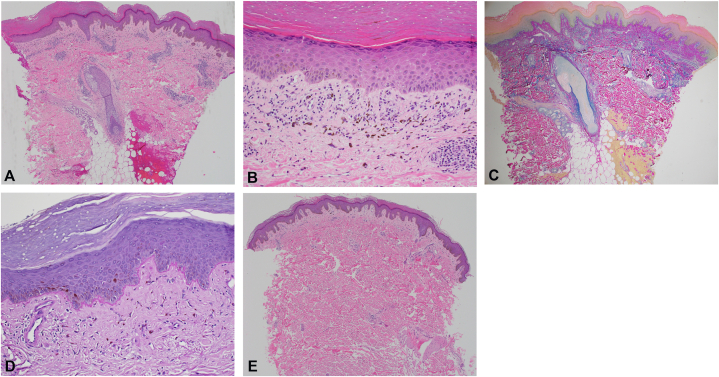
Histology. Right periumbilical, hematoxylin and eosin **(A)** 4× and **(B)** 20×, showing lichenified interface dermatitis with increased dermal mucin. **C,** Right periumbilical, 4×, colloidal iron stains highlight increased dermal mucin throughout the reticular dermis. **D,** Right periumbilical, 20×, PAS stain showed a focally thickened basement membrane. **E,** Left lower back, hematoxylin and eosin 4×, showing hyperkeratosis and epidermal hyperplasia with subtle fibrosis of the superficial dermis and numerous melanophages, with rare necrotic keratinocytes, consistent with erythema ab igne with lichen simplex chronicus. *PAS*, Periodic acid–Schiff.

Histopathology of the periumbilical region revealed hyperkeratosis, acanthotic epidermis, vacuolar alteration at the dermo-epidermal junction, a focally thickened basement membrane, superficial dermal fibrosis, and a superficial and mid-dermal perivascular and lymphohistiocytic inflammatory cell infiltrate. Colloidal iron stains revealed increased dermal mucin throughout reticular dermis (Fig 2). The findings were read as a lichenified interface dermatitis with increased dermal mucin, consistent with DLE and superimposed lichen simplex chronicus. Periodic acid–Schiff staining showed a focally thickened basement membrane, and colloidal iron staining indicated increased dermal mucin throughout the reticular dermis. The lower back biopsy showed hyperkeratosis and epidermal hyperplasia with subtle fibrosis of the superficial dermis and numerous melanophages, with rare necrotic keratinocytes (Fig 2). Findings were consistent with erythema ab igne with lichen simplex chronicus changes. Antinuclear antibody was positive with a titer of 1:1280 in a speckled pattern. Complete blood count, metabolic panel, complement component 4, complement component 3, and urinalysis were normal. Double-stranded DNA antibody was negative. The patient was started on ruxolitinib cream twice daily and clobetasol cream as needed, along with cetirizine for itch, daily sunscreen, and counseling to refrain from scratching. He was also continued on oral hydroxychloroquine and anifrolumab infusions. Despite ongoing therapy, the extracephalic lesions persisted. The patient did not discontinue the heating blanket until 4 months after initial presentation, after which the lesions gradually resolved and the itching subsided at subsequent follow-up visits.

## Discussion

This case represents an unusual instance of heat-induced DLE due to the Koebner phenomenon. While ultraviolet radiation is a known trigger for lupus, heat as a trigger is uncommon. Our patient experienced lesions on face and neck consistent with typical DLE, and then developed new lesions on trunk related to heat exposure.

The Koebner phenomenon refers to lesion development at sites of trauma in patients with an underlying history of the same dermatosis.^2^ The proposed mechanism is a 2-step process: an environmental factor such as heat induces an inflammatory response, which then activates a condition-specific inflammatory process at the site of injury.^3^

Koebner phenomenon is well described in psoriasis and lichen planus but is rarely reported in cutaneous lupus. External irritants have been recognized as triggers for DLE since 1926, including tattoos,^4^ an oil scald,^5^ burns,^6^ scars,^7^ and only 2 due to chronic heat.^8^^,^^9^ One case describes heat-induced DLE in a 63-year-old woman with SLE, presenting with chronic lesions on her back and thumb due to prolonged exposure to hot ovens and stirring pots as a cook.^8^ She also exhibited a reticular hyperpigmented plaque on her back resembling erythema ab igne, but histopathology was consistent with DLE.^8^ The authors first proposed the term “lupus ab-igne,” meaning “lupus from fire” in Latin. The second case presents a 34-year-old woman with SLE and Sjogren syndrome who uses electronic cigarettes and developed histology-confirmed, isolated DLE lesion on her upper lip.^9^ The lesion did not resolve with continued smoking, further substantiating the role of chronic heat exposure as a trigger.

Our patient developed DLE lesions on the chest and back after years of heating blanket use. While the lesions could represent chronic DLE, the atypical distribution and subsequent resolution upon cessation of heating blanket use better support Koebnerization. Notably, the patient had used both ruxolitinib and clobetasol for the extracephalic lesions for 4 months without improvement. Despite these agents having previously demonstrated efficacy for his cephalic DLE, the extracephalic lesions persisted until the patient discontinued the heated blanket approximately 4 months after initial presentation, suggesting that removal of the thermal stimulus was a key intervention, although ruxolitinib and clobetasol may have worked in tandem as a contributing intervention. There was also separate histopathologic evidence of erythema ab igne on the lower back, lacking overlapping features of DLE, which may suggest a predisposition to heat-triggered inflammatory reactions. Curiously, the erythema ab igne did not exhibit the characteristic reticular pattern typically seen in other reports.^8^ Wolf’s isotopic phenomenon, the occurrence of an unrelated dermatosis at the site of a previously healed disease,^10^ was considered. However, there was no clinical or histological evidence of a preceding dermatosis at the sites where DLE developed, although we cannot definitively exclude a prior process that resolved before presentation. Overall, although trauma is known to trigger DLE, it is often overlooked during prevention and management. This case emphasizes caution with prolonged exposure to low-grade, continuous heat sources in patients with cutaneous lupus.

## Conflicts of interest

None disclosed.
